# Erector Spinae Plane Block Versus Thoracic Paravertebral Block in Laparoscopic Cholecystectomy: A Randomized Controlled Study

**DOI:** 10.3390/jcm15124593

**Published:** 2026-06-13

**Authors:** Özlem Turhan, Zerrin Sungur, Müşerref Beril Dinçer, Meltem Savran Karadeniz, Esra Saka, Hacer Ayşen Yavru, Reyhan Nil Kırşan, Nükhet Sivrikoz

**Affiliations:** Department of Anesthesiology and Reanimation, İstanbul Faculty of Medicine, İstanbul University, İstanbul 34093, Türkiye; ozlemturhan6@gmail.com (Ö.T.);

**Keywords:** erector spinae plane block, paravertebral block, postoperative pain, quality of recovery, laparoscopic cholecystectomy

## Abstract

**Objectives**: This randomized, single-blind study aimed to compare the effects of ultrasound-guided erector spinae plane block (ESPB), thoracic paravertebral block (TPVB) and intravenous (IV) analgesia on postoperative pain, opioid consumption and quality of recovery in patients undergoing laparoscopic cholecystectomy (LC). **Methods**: A total of 120 adult patients (ASA I-III) scheduled for elective LC were randomized into three groups: ESPB (GI), TPVB (GII) and IV analgesia (GIII). Bilateral ESPB or TPVB was performed preoperatively; then all patients received standardized general anesthesia and postoperative analgesia including paracetamol, tenoxicam and IV tramadol via patient-controlled analgesia. The primary outcome was 24 h tramadol consumption. Secondary outcomes included pain scores, rescue analgesia requirement, patient satisfaction, postoperative nausea and vomiting, time to first ambulation, length of hospital stay and Quality of Recovery-15 (QoR-15) scores. **Results**: Twenty-four-hour tramadol consumption was significantly higher in GIII (135.78 ± 22.73 mg) compared with GI (101.05 ± 26.99 mg) and GII (95.67 ± 31.49 mg) (*p* < 0.001), with no difference between GI and GII. Both static and dynamic pain scores were lower in GI and GII compared with GIII at most time points. Rescue analgesia requirement and patient dissatisfaction were significantly higher in GIII. QoR-15 scores were significantly improved in GI and GII compared with GIII (*p* < 0.001), while no difference was observed between the regional techniques. Block performance time was shorter with ESPB than TPVB (*p* < 0.001). No complications were reported. **Conclusions**: ESPB and TPVB provided effective analgesia and improved recovery after LC compared with IV analgesia alone. Both regional techniques may be considered as components of multimodal analgesia after LC.

## 1. Introduction

Laparoscopic cholecystectomy (LC) has largely replaced open surgery due to its well-recognized advantages, including reduced surgical trauma, faster recovery, and shorter hospital stay [1]. Although LC is less invasive procedure, post-surgical pain continues to be a relevant issue that can negatively impact patient comfort and recovery [2,3].

The pain after LC includes somatic pain arising from trocar entry sites, as well as visceral pain resulting from peritoneal stretching, pneumoperitoneum and diaphragmatic irritation [3,4]. This complex pain profile necessitates a multimodal analgesia approach to optimize postoperative outcomes. Opioids and non-steroid anti-inflammatory drugs (NSAID) are frequently used for postoperative analgesia; however, their use may be limited by dose-dependent adverse effects such as respiratory depression, nausea, vomiting, renal dysfunction and potential for dependency [5]. Consequently, regional analgesia techniques have gained increasing attention as part of multimodal analgesia strategies, particularly with the widespread use of ultrasound (US) guidance, which has improved the safety and success rate of these techniques.

Thoracic paravertebral block (TPVB) is a well-established regional analgesia technique that provides effective somatic and visceral analgesia [6,7,8]. However, concerns about complications related to its proximity to the pleura may discourage its use. Erector spinae plane block (ESPB) has become a promising alternative regional analgesia technique owing to its technical simplicity and low complication profile [3,9]. It involves administering local anesthetic (LA) into the fascial plane located beneath the erector spinae muscle, allowing cranio-caudal spread and possible extension into the paravertebral space [10]. Clinical studies have reported encouraging results in reducing postoperative pain in various surgical procedures [11,12]. Recent experimental data suggest that LA spread in fascial plane blocks may also involve anisotropic diffusion through muscle and fascial tissues, which may not be fully captured by conventional imaging or dye-based models [13].

Despite increasing interest in both ESPB and TPVB, comparative studies evaluating these techniques in LC remain scarce. Existing studies have primarily focused on postoperative opioid consumption and pain scores, whereas outcomes focusing on patient experience, including quality of recovery, have not been adequately investigated. Therefore, further evidence is needed to clarify their analgesic efficacy as well as patient-centered outcomes.

To the best of our knowledge, while randomized trials have compared ESPB and TPVB with respect to analgesic outcomes, no study has evaluated both analgesic efficacy and quality of recovery in patients undergoing LC. The present randomized trial was designed to compare the effects of US-guided ESPB and TPVB with a control group regarding postoperative analgesia and quality of recovery in patients undergoing LC.

## 2. Materials and Methods

### 2.1. Study Design

This randomized, single-blind trial was approved by the Istanbul University Istanbul Faculty of Medicine Clinical Research Ethics Committee (2025/1127). The study was registered at Clinical-Trials.gov (registration number: NCT07149584) prior to study initiation. This study was conducted and reported in line with the CONSORT guidelines for randomized controlled studies.

The study included adult patients (≥18 years) scheduled for elective LC between September 2025 and February 2026 with an American Society of Anesthesiologists (ASA) physical status I-III. Exclusion criteria included chronic use of analgesics, contraindications to regional anesthesia (e.g., coagulopathy, ongoing anticoagulant therapy, hypersensitivity to LA), and procedures requiring open cholecystectomy or intraoperative conversion to an open approach. All patients signed informed consent after being informed about the study.

Patients were allocated to Group I (GI), Erector Spinae Plane Block; Group II (GII), Thoracic Paravertebral Block; and Group III (GIII), Intravenous (IV) Analgesia, according to the analgesic technique used. Group allocation was determined using a computer-generated randomization list, with group assignments concealed in sealed opaque envelopes that were opened only by the anesthesiologists performing the blocks. The anesthesiologists who performed the blocks were not involved in perioperative management or data collection. Staff anesthesiologists responsible for perioperative management and outcome evaluators were unaware of group allocation.

### 2.2. Regional Analgesia Procedures

After standard monitoring according to ASA recommendations in the operating room, 2 mg of IV midazolam was administered for sedation. The T8 vertebral level was identified using anatomical landmarks; the inferior angles of the scapula were used as a reference corresponding to the T7 level, and the T8 level was determined by counting caudally from the C7 spinous process by palpation. One of the experienced anesthesiologists specialized in regional anesthesia (Ö.T. or N.S.) performed the blocks (ESPB or TPVB) bilaterally at the T8 level under US guidance to the patient in the sitting position. Both blocks were performed prior to induction of general anesthesia using a linear probe oriented in a longitudinal plane with an out-of-plane needle insertion technique. For ESPB, 10 mL of 0.375% bupivacaine was injected into the fascial plane beneath the erector spinae muscle on each side. Block success was confirmed by shifting the US probe cranially to the T6-T7 levels and caudally to the T9-T10 levels to visualize the interfascial spread of LA. For TPVB, 10 mL of 0.375% bupivacaine was administered on each side into the paravertebral space. Similarly, block success was confirmed by moving the US probe cranially and caudally to observe distribution of LA above the pleura. The duration of block performance was recorded separately for each side and was defined as the time from the placement US probe on the block area to the needle withdrawal. The total block performance time was calculated as the sum of the durations recorded for both sides.

### 2.3. Perioperative Anesthesia and Surgical Technique

General anesthesia was standardized across all study groups using IV fentanyl (2 μg/kg) and propofol (2 mg/kg) followed by neuromuscular blockade with rocuronium (0.5 mg/kg) for induction and sevoflurane inhalation was used for maintenance. All patients received IV dexamethasone (8 mg) and ondansetron (4 mg) for prophylaxis of postoperative nausea and vomiting (PONV). Hypotension (a > 20% decrease in blood pressure from baseline or a mean arterial pressure < 65 mmHg) was managed with a norepinephrine infusion, and bradycardia (a heart rate < 50 bpm) with IV atropine.

All patients underwent LC in the supine position using a standard four-port technique. Pneumoperitoneum was established with carbon dioxide at an intraabdominal pressure of 12–14 mmHg. The trocars were placed according to the conventional four-port approach, including an umbilical camera port and additional working ports in the epigastric and right subcostal regions. The trocars were removed under direct visualization, and surgical incisions were closed in a standard manner at the end of the procedure.

### 2.4. Analgesia Protocol and Postoperative Follow-Up

As a part of multimodal analgesia regimen, all patients received IV paracetamol (1 gr, three times daily) and tenoxicam (20 mg, once daily) with the first doses administered intraoperatively. Postoperative analgesia was standardized for all study groups and consisted of patient-controlled analgesia (PCA) with IV tramadol (bolus dose: 20 mg and lockout interval: 20 min). The primary outcome was 24 h postoperative tramadol consumption.

Pain intensity was evaluated both at rest (static) and on coughing (dynamic) using the numeric rating scale (NRS; 0 indicates no pain and 10 represents the worst imaginable pain) at 0, 1, 4, 6, 12, 18 and 24 h after surgery. Rescue analgesia with IV meperidine (0.5 mg/kg) was given whenever NRS was above 3 at any assessment time point.

Time to first ambulation, shoulder pain and block-related complications (vascular puncture, pneumothorax, hematoma and hypotension) were recorded along with length of hospital stay (LOS). If PONV occurred despite intraoperative prophylaxis, 10 mg metoclopramide was administered as rescue therapy. Patient satisfaction with the postoperative analgesia management was rated using three-point Likert scale (1 = satisfied, 2 = neutral, 3 = unsatisfied).

Quality of postoperative recovery was evaluated at 24 h or prior to discharge using the validated Turkish version of the Quality of Recovery-15 (QoR-15) questionnaire which yields a total score extending from 0 (extremely poor recovery) to 150 (excellent recovery) [14,15].

### 2.5. Statistical Analysis

The sample size was calculated based on 24 h postoperative tramadol consumption in patients who received ESPB, TPVB or IV analgesia in a pilot study including 10 patients in each group. Tramadol consumption was 98 ± 23.82 mg in patients who received ESPB, 88 ± 22.38 mg in those who received TPVB and 120 ± 24.94 mg in those who received IV analgesia. The required sample size was calculated to be at least 36 patients per group, assuming an alpha level of 0.05 and a power of 80% (beta:0.20). Considering potential dropouts, a total of 120 patients were included in the study.

The distribution of the data was assessed using the Kolmogorov–Smirnov test. Normally distributed variables were presented as mean ± standard deviation whereas non-normally distributed data were presented as median (minimum-maximum). Frequencies and percentages were used to describe categorical variables, which were analyzed with the chi-square test. Pairwise comparisons were performed using the chi-square test with Bonferroni correction. Normally distributed variables were compared among the three groups using one-way analysis of variance (ANOVA). In cases where one-way ANOVA indicated statistical significance, Bonferroni-corrected post hoc tests were applied for pairwise comparisons, with *p* < 0.017 considered statistically significant. Non-normally distributed variables were compared using the Kruskal–Wallis test. When a statistically significant difference was detected, pairwise comparisons were performed using Mann–Whitney U tests Bonferroni correction. All analyses were conducted using IBM SPSS, 21.0 (IBM Corp, Armonk, NY). Statistically significance was set at *p* <0.05.

## 3. Results

**Figure 1:**
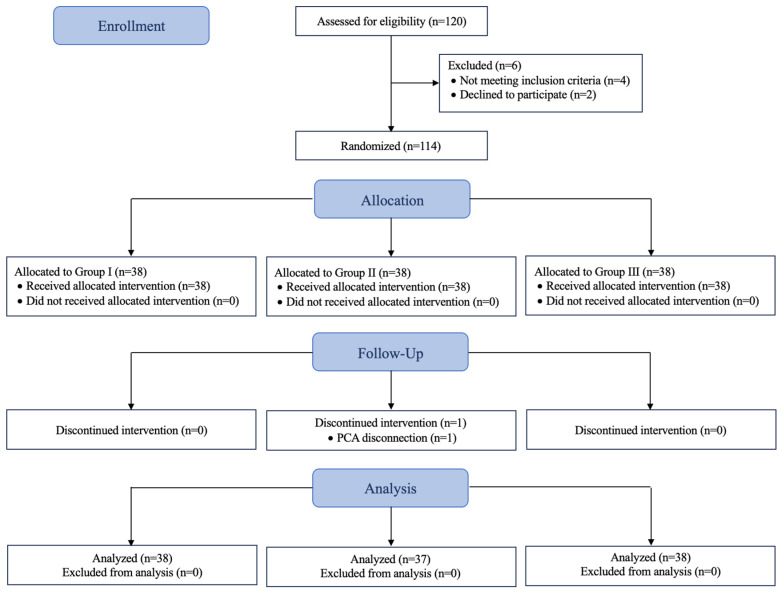
CONSORT flow diagram.

In this study, 120 patients scheduled for elective LC were enrolled. Seven patients were excluded, as outlined in the flow diagram (Figure 1). Finally, 113 patients were included in the analysis: 38 in GI, 37 in GII and 38 in GIII. Demographic and perioperative characteristics were similar among groups (Table 1).

There was a significant difference in 24 h postoperative tramadol consumption across the study groups (*p* < 0.001). Mean tramadol consumption was 101.05 ± 26.99 mg in GI, 95.67 ± 31.49 mg in GII and 135.78 ± 22.73 mg in GIII. Post hoc analysis demonstrated significantly higher consumption in GIII compared with GI (mean difference 34.73 mg, 95% CI: 17.08 to 52.39; *p* < 0.001) and GII (mean difference 40.11 mg, 95% CI: 22.34 to 57.88; *p* < 0.001). Comparison of GI and GII revealed no statistically significant difference (*p* = 1).

Static NRS scores differed significantly among the groups at 0, 1, 4, 6, 12 and 18 h (*p* < 0.05), whereas no significant difference was observed at 24 h (*p* > 0.05). At all follow-up time points, dynamic NRS scores demonstrated statistically significant differences across the study groups (*p* < 0.05). Detailed intergroup NRS scores and statistical comparisons are presented in Table 2. Rescue analgesia requirement was as follows: three patients (7.8%) in GI (at 0, 1 and 12 h), three patients (8.1%) in GII (at 1 and 6 h), and 12 patients (31.5%) in GIII (at 0, 1, 4, 6 and 12 h), with a statistically significant intergroup difference (*p* = 0.005). Post hoc analysis demonstrated that the requirement for rescue analgesia was significantly lower in both GI and GII compared with GIII (*p* = 0.009 and *p* = 0.011), while it was comparable in GI and GII (*p* = 0.97).

Postoperative shoulder pain was reported in four patients (10.5%) in GI, three (8.1%) in GII and five (13.1%) in GIII. Patients with NRS scores >3 for shoulder pain received rescue analgesia: one patient in GI at 12 h, two in GII at 6 h, and two in GIII at 6 and 12 h. Shoulder pain experience was comparable between the groups (*p* = 0.76).

The total block performance time was shorter in GI than in GII (185.36 ± 37.66 s vs. 247.4 ± 31.13 s, *p* < 0.001). Thirty-three patients (86.8%) in GI were satisfied with the postoperative analgesia management (three were neutral, two were unsatisfied), 30 patients (81.1%) in GII were satisfied (three were neutral, four were unsatisfied) and 23 patients (60.5%) in GIII were satisfied (seven were neutral, eight were unsatisfied). Patient satisfaction with postoperative analgesia was significantly higher in GI and GII compared with GIII (*p* = 0.004 and *p* = 0.013, respectively). No significant difference was observed between GI and GII (*p* = 0.7).

Four patients in GI, five patients in GII and seven patients in GIII experienced PONV and they were treated with 10 mg metoclopramide (*p* = 0.6). No complications were observed in any patients. Time to first ambulation and LOS were comparable among the groups (Table 1).

Mean global QoR-15 scores were 131.44 ± 6.25 in GI, 128.56 ± 5.29 in GII and 122.97 ± 6.07 in GIII (*p* < 0.001). Patients’ global QoR-15 scores were higher in GI and GII compared with GIII (*p* < 0.001, both), whereas scores were comparable between GI and GII (*p* = 0.110). When the individual QoR-15 domains were analyzed (Table 3), emotional state and psychological support domains showed no significant intergroup differences. Pain domain scores were similar between GI and GII, but significantly higher in both groups compared with GIII (*p* < 0.001 and *p* < 0.001, respectively). Physical comfort scores showed a similar pattern, being comparable between GI and GII but significantly higher in both than in GIII (*p* < 0.001 and *p* < 0.001, respectively). Physical independence scores were also similar between GI and GII, while both groups had significantly higher scores compared with GIII (*p* < 0.001 and *p* = 0.011, respectively).

## 4. Discussion

This study demonstrated that ESPB and TPVB markedly reduced postoperative opioid consumption and provided superior analgesia with lower pain scores than IV systemic analgesia in patients undergoing LC. Besides better pain control, regional methods resulted in better recovery scores compared to IV analgesia alone. Moreover, both regional blocks were also associated with greater patient satisfaction.

Though widely performed and associated with considerable postoperative pain, analgesia management in LC is yet to be defined. Recently, procedure-specific postoperative pain management (PROSPECT) recommended basic analgesia and favored LA infiltration techniques over regional methods due to concerns regarding systemic LA toxicity [2]. However, previous studies have shown that both ESPB and TPVB provide better pain control than systemic analgesia, as reflected by reduced opioid requirements [16,17,18,19,20,21,22]. Furthermore, a meta-analysis on this topic has suggested a variety of regional block techniques depending on the outcomes [23].

In the search for optimal analgesia management in LC, a recent study compared ESPB and TPVB with the systemic analgesia approach recommended by PROSPECT guidelines [24]. In this context, TPVB showed significantly better analgesic efficacy with lowest tramadol consumption compared to ESPB and systemic analgesia. The authors explained that the relatively lower analgesic efficacy of ESPB might be attributable to its unilateral application, while they did not consider the unilateral application of TPVB. Authors also reported better pain scores in TPVB than in control group. Our study differs from the previous one in terms of bilateral injection for both ESPB and TPVB as well as LA concentration (0.25% vs. 0.375%). As the primary outcome, we observed higher tramadol consumption in IV analgesia group compared with ESPB and TPVB whereas there was no difference between the two block techniques. For patients undergoing LC, the minimum clinically important difference (MCID) in opioid consumption for postoperative analgesic outcomes has not been clearly established. Although a reduction of approximately 5 mg intravenous morphine equivalent during the first 24 postoperative hours has been proposed as a clinically meaningful threshold, this estimate was derived from heterogeneous studies reporting a wide range of opioid differences, including smaller reductions [25]. A recent review found insufficient evidence to define a clear MCID for 24 h postoperative opioid consumption and emphasized the need for more comprehensive patient-centered outcome frameworks in future research [26]. In the present study, the between-group difference in opioid consumption corresponded to approximately 35–40 mg of tramadol. This opioid-sparing effect was also accompanied by lower pain scores, reduced rescue analgesic requirements, higher patient satisfaction, and improved quality-of-recovery outcomes. Therefore, the observed benefit may reflect a clinically perceptible improvement in the overall postoperative recovery experience rather than a reduction in opioid consumption alone.

Bezen et al. using the same regional protocol (unilateral, 0.25% bupivacaine), found comparable analgesic efficacy for ESPB and TPVB in terms of pain scores and rescue analgesia requirements, and their results are consistent with our findings [27]. In fact, both studies reported similar pain scores for regional blocks [24,27]. The main difference in their methodology lies in postoperative pain management, where, after a single dose of NSAID, analgesia was based on rescue medication stratified according to pain intensity (lower pain scores treated with non-opioids, higher scores with different opioids) [27]. This approach obviously complicates a more objective comparison as underlined in the limitations of the study. In our study, all groups achieved effective analgesia, while control group required a significantly higher dose of tramadol and the number of patients requiring rescue analgesia was also significantly higher. We can therefore suggest that bilateral block application and LA concentration were associated with more effective analgesia. The issue of concentration has been previously discussed, and investigators have proposed an increased concentration to avoid block failure in ESPB for LC analgesia [20]. The use of a 0.25% LA solution with a 20 mL volume was associated with a higher incidence of block failure and insufficient sensory blockade, which was attributed to inadequate spread within the fascial plane. Therefore, in the present study, a similar concentration and volume strategy (20 mL 0.375%) was adopted in light of these findings to ensure adequate spread while maintaining an acceptable safety profile. In line with this, bilateral ESPB has been associated with better pain control not only compared with systemic analgesia but also compared with unilateral block [28].

The perioperative care—especially in minor surgery—is intended to ensure good and early recovery. Patient-reported outcome questionaries are reliable tools in this regard, assessing different domains. We used the QoR-15 questionnaire, which is a revised brief form of QoR-40, to assess postoperative recovery [14,15]. To our knowledge, this is the first study comparing quality of recovery among ESPB, TPVB and a control group in LC. In this study, ESPB and TPVB groups resulted in significantly better QoR-15 scores compared with systemic analgesia group (131.44, 128.56 and 119.1, respectively). Moreover, according to the classification proposed by Kleif et al., the two regional analgesia groups in the present study were categorized as having good recovery, whereas the control group was classified as having moderate recovery [29]. In fact, clinically meaningful change for QoR-15 has been defined as a difference of 6 point, and both regional techniques ensured the difference [30]. When focusing on the domains of QoR-15, the observed differences were, as expected, by improvements in pain, as well as in physical comfort and in physical independence, probably related to better pain control. Similarly, ESPB alone, compared with systemic multimodal analgesia, was associated with improved quality of recovery [31]. Using QoR-40 questionnaire, authors reported a significant minimal clinically important difference. Compared to port-site injection and systemic analgesia, ESPB resulted in higher QoR-15 scores among LC patients [32]. Functional recovery was better in all domains for ESPB group than for the control group.

Focused on other recovery parameters, time to first ambulation and LOS were comparable across the study groups, as described in the literature [16,24,27]. The lack of difference in time to first ambulation and LOS despite higher QoR-15 scores may be attributed to the fact that these outcomes are largely influenced by standardized institutional discharge protocols and perioperative pathways rather than analgesia alone. In contrast, QoR-15 is a multidimensional patient-reported outcome measure that is more sensitive to differences in pain, comfort, and overall well-being. Therefore, improvements in subjective recovery scores may not necessarily translate into changes in protocol-driven objective endpoints in this setting. Postoperative nausea and vomiting incidence were similar among all groups, consistent with the existing literature; this may be attributable to anti-emetic prophylaxis administered to all patients [21,27]. Shoulder pain was also comparable across all groups [20,24,27].

Regarding block performance time, Bezen et al. observed longer durations for TPVB compared with ESPB without statistical significance [27]. In a meta-analysis comparing ESPB and TPVB in breast and thoracic surgeries, duration of block performance time for ESPB was reported to be significantly shorter than that for TPVB, which was also observed in our study [33]. This difference may be related to the technical advantages of ESPB, due to its relative simplicity.

Literature on patient satisfaction with ESPB and TPVB in LC is limited and shows variable results. Mandal et al. found no significant difference between ESPB and control groups, with dissatisfaction observed only in the control group [21]. A comparative study reported higher satisfaction with TPVB than with ESPB, although the difference (4.49 vs. 4.14) on a five-point Likert scale was minimal [27]. Yılmaz et al. stated higher satisfaction in both ESPB and TPVB compared with the control group [24]. In our study, regional blocks resulted in higher patient satisfaction compared with IV analgesia which may be explained by lower pain scores and reduced opioid need.

Regional methods are potentially prone to systemic complications that may affect their use in LC analgesia, particularly those performed in proximity of neuroaxial structures and pleura. Notably, ESPB has been associated with a favorable safety profile, with no block-related complications reported in several studies [20,21,22,27]. In this study, we did not observe block-related complications in either group. It should be noted that our primary outcome was postoperative opioid consumption rather than complication rates.

The limitations of this study should be considered. Firstly, sensory dermatomal blockade was not evaluated; however, all blocks were performed by experienced anesthesiologists, and block success was assessed based on the spread of LA under US guidance. Nevertheless, dermatomal distribution of the block may vary between individuals and plays an important role in the clinical interpretation of analgesic efficacy. Therefore, the absence of dermatomal assessment limits a more detailed mechanistic comparison between ESPB and TPVB, despite the observed comparable analgesic outcomes. Secondly, quality of recovery was measured using the QoR-15 only in the postoperative period. Since patients with acute cholecystitis were excluded and the study population consisted of asymptomatic patients scheduled for elective surgery, baseline differences in preoperative recovery status were expected to be minimal; therefore, a preoperative QoR-15 assessment was not included in the study design. Lastly, the study was conducted as a single-blind trial comparing regional block techniques with a control group receiving IV analgesia only. A sham block group was not included, which may have introduced a potential placebo-related bias. Nevertheless, the study design was appropriate to evaluate the effectiveness of the blocks compared with standard IV analgesia, which was the primary objective of the study.

## 5. Conclusions

Erector spinae plane block and TPVB were associated with favorable analgesic outcomes and improved quality of recovery after LC compared with systemic analgesia alone. Postoperative analgesic outcomes appeared comparable between the two regional techniques. Accordingly, both ESPB and TPVB may be considered as components of multimodal analgesia after LC.

## Figures and Tables

**Table 1:** Demographic and perioperative data of the patients.

	GI (*n* = 38)	GII (*n* = 37)	GIII (*n* = 38)	*p* Value
Age (years)	51.44 ± 15.55	51.51 ± 14.1	51.81 ± 15.37	0.994
Height (cm)	165.89 ± 8.14	167.89 ± 8.59	165.55 ± 9.28	0.456
Weight (kg)	73.78 ± 12.02	74.51 ± 10.94	74.55 ± 12.76	0.952
Gender				0.971
Female	20 (52.63%)	18 (48.64%)	19 (50%)	
Male	18 (47.36%)	19 (51.35%)	19 (50%)	
ASA status				0.982
I	14 (36.84%)	14 (37.83%)	16 (42.1%)	
II	18 (47.36%)	19 (51.35%)	20 (52.63%)	
III	6 (15.78%)	4 (10.81%)	5 (13.15%)	
Duration of anesthesia (min)	75 (55–125)	75 (60–120)	75 (50–130)	0.742
Duration of surgery (min)	65 (50–115)	65 (50–105)	62.5 (40–115)	0.753
Time to first ambulation (h)	6 (4–9)	7 (4–9)	6 (4–10)	0.402
Length of hospital stay (h)	25.44 ± 1.91	25.72 ± 2.03	25.71 ± 1.43	0.751

ASA, American Society of Anesthesiologists; GI, Group I—Erector Spinae Plane Block; GII, Group II—Thoracic Paravertebral Block; GIII, Group III—Intravenous Analgesia. Data are presented as mean ± SD or *n* (%). *p* values were calculated using one-way ANOVA, the Kruskal–Wallis test, the chi-square test, as appropriate.

**Table 2:** Postoperative NRS scores.

	GI (*n* = 38)	GII (*n* = 37)	GIII (*n* = 38)
0th min			
Static	3 (1–4) *	3 (2–5) ^#^	3 (2–6)
Dynamic	4 (3–6) *	4 (4–5) ^#^	5 (3–7)
1st h			
Static	2 (2–4) *	2 (2–4) ^#^	3 (2–5)
Dynamic	3 (2–4) *	3 (2–4) ^#^	4 (3–6)
4th h			
Static	2 (0–3) *	2 (0–3) ^#^	3 (2–4)
Dynamic	3 (2–4) *	3 (2–4) ^#^	3 (2–6)
6th h			
Static	2 (0–2) *	2 (0–5) ^#^	2 (2–6)
Dynamic	2 (0–3) ^#^	2 (0–3) ^#^	3 (0–5)
12th h			
Static	0 (0–4) *	0 (0–1) ^#^	2 (0–5)
Dynamic	2 (0–2) *	0(0–2) ^#^	2 (0–3)
18th h			
Static	0 (0–1) *	0 (0–1) ^#^	0 (0–2)
Dynamic	0 (0–2) *	0 (0–2) ^#^	0 (0–3)
24th h			
Static	0 (0–0)	0 (0–0)	0 (0–1)
Dynamic	0 (0–0) *	0 (0–0) ^#^	0 (0–2)

GI, Group I—Erector Spinae Plane Block; GII, Group II—Thoracic Paravertebral Block; GIII, Group III—Intravenous Analgesia; NRS, numeric rating scale. Data are presented as median (minimum-maximum). *p* values were calculated using the Mann–Whitney U test, and *p* < 0.017 is accepted for statistical significance with Bonferroni adjustment. * *p* < 0.017 compared with Group III. ^#^ *p* < 0.017 compared with Group III.

**Table 3:** QoR-15 domains.

		GI (*n* = 38)	GII (*n* = 37)	Group III (*n* = 38)
Physical well-being	Physical comfort	45.55 ± 3.29 *	44.86 ± 3.09 **^#^**	40.15 ± 3.99
	Physical independence	17.15 ± 1.55 *	16.4 ± 2 **^#^**	14.5 ± 2.02
	Pain	16.02 ± 3.01 *	16.13 ± 2.34 **^#^**	12.47 ± 1.89
Mental well-being	Psychological support	17.84 ± 2.09	17.37 ± 1.75	17.21 ± 2.24
	Emotional state	34.86 ± 1.94	33.78 ± 2.81	34.76 ± 2.09

GI, Group I—Erector Spinae Plane Block; GII, Group II—Thoracic Paravertebral Block; GIII, Group III—Intravenous Analgesia; QoR, Quality of Recovery. Data are presented as mean ± SD. *p* values were calculated using one-way ANOVA, and *p* < 0.017 is accepted for statistical significance with Bonferroni adjustment. * *p* < 0.017 compared with Group III. ^#^ *p* < 0.017 compared with Group III.

## Data Availability

The data presented in this study are available on reasonable request from the corresponding author.

## Abbreviations

The following abbreviations are used in this manuscript:

LC	laparoscopic cholecystectomy
NSAID	non-steroid anti-inflammatory drugs
US	ultrasound
TPVB	thoracic paravertebral block
ESPB	erector spinae plane block
LA	local anesthetic
ASA	American Society of Anesthesiologists
GI	Group I
GII	Group II
GIII	Group III
IV	intravenous
PONV	postoperative nausea and vomiting
PCA	patient-controlled analgesia
NRS	numeric rating scale
LOS	length of hospital stay
QoR	quality of recovery
PROSPECT	procedure-specific postoperative pain management
MCID	minimum clinically important difference
